# Artificial Pancreas and Continuous Insulin Infusion for Preventing Cardiac Surgical Site Infections

**DOI:** 10.1093/ejcts/ezag166

**Published:** 2026-06-06

**Authors:** Takeshi Arai, Daichi Takagi, Hiroshi Yamamoto, Hiroyuki Nakajima

**Affiliations:** Department of Cardiovascular Surgery, Akita University Graduate School of Medicine, Akita 010-8543, Japan; Department of Cardiovascular Surgery, Akita University Graduate School of Medicine, Akita 010-8543, Japan; Medical DX Center, Division of Telemedical Promotion, Akita University Graduate School of Medicine, Akita 010-8543, Japan; Department of Cardiovascular Surgery, Akita University Graduate School of Medicine, Akita 010-8543, Japan

**Keywords:** surgical site infection, artificial pancreas, glycaemic control, cardiovascular surgery

## Introduction

Surgical site infections (SSIs) complicate 3%-10% of cardiac surgeries and increase morbidity and mortality.^1^^,^^2^ Stress-induced acute hyperglycaemia impairs immune function and exacerbates inflammation, making effective glycaemic management critical.^3^ Beyond absolute blood glucose (BG), optimizing time in range (TIR) and minimizing glycaemic variability (coefficient of variation, CV) significantly influence outcomes.^4^^,^^5^ While European Association for Cardio-Thoracic Surgery (EACTS) guidelines recommend postoperative glucose <180 mg/dL,^6^ standard continuous insulin infusion (CII) relies on intermittent sampling and reactive adjustments, often resulting in suboptimal TIR and increased hypoglycaemia risk. Conversely, an artificial pancreas (AP) system enables continuous, automated minute-by-minute modulation.^7^ We hypothesized that an ICU-based automated AP system would improve metabolic stability and reduce SSI incidence compared with CII in patients undergoing cardiopulmonary bypass (CPB).

## Methods

### Study design and population

This retrospective study analysed 208 adults undergoing cardiac surgery with CPB at Akita University Hospital (2021-2022). After excluding patients with pre-existing infections or delayed insulin initiation, participants were grouped according to the 48-hour ICU glycaemic protocol: the AP group (*n* = 91) and CII group (*n* = 117), determined by device availability.

### Blood glucose management

The AP group utilized the STG-55 closed-loop system (Nikkiso Co., Ltd.), which measures BG every minute, automatically adjusting infusions targeting 100-120 mg/dL. The CII group followed institutional protocols targeting <180 mg/dL with 3-hourly manual BG measurements and sliding-scale adjustments.

### Outcomes and statistical analysis

The primary outcome was the incidence of SSIs (CDC/NHSN criteria).^8^ Secondary endpoints included mean BG, CV, TIR (70-180 mg/dL), and time to BG <180 mg/dL. To mitigate selection bias, 1:1 propensity score matching (calliper 0.2) balanced 13 baseline covariates (e.g., age, sex, BMI, diabetes, procedure, CPB time, BG levels at start of surgery and admission to ICU) (Table 1).

**Table 1.** Baseline Characteristics and Outcomes From Each Group Pre- and Post-Matching

	Before matching			After matching			
Characteristics	CII (*n* = 117)	AP (*n* = 91)	*P*-value	CII (*n* = 66)	AP (*n* = 66)	*P*-value	SMD
Age (year)	70 [64-76]	72 [62-76]	.951	68 [64-75]	72 [62-76]	.529	0.113
Sex male, *n* (%)	65 (55.6)	68 (74.7)	.007	46 (69.7)	47 (71.2)	>.99	0.033
Body mass index (kg/m^2^)	23.5 [20.8-26.6]	22.7 [21.4-26.6]	.644	22.9 [20.5-25.4]	22.9 [21.4-26.6]	.980	0.098
Diabetes, *n* (%)	33 (28.2)	27 (29.7)	.93	19 (28.8)	20 (30.3)	>.99	0.033
Insulin-dependent, *n* (%)	6 (5.1)	7 (7.7)	.64	4 (6.1)	6 (9.1)	.742	0.115
Haemodialysis, *n* (%)	9 (7.7)	6 (6.6)	.97	5 (7.6)	5 (7.6)	>.99	<0.001
Preoperative HbA1c (%)	5.9 [5.5-6.2]	5.8 [5.5-6.2]	.824	–	–	–	–
Surgical procedures							
Main procedures			.001			.84	0.159
Aorta, *n* (%)	59 (50.4)	24 (26.4)		21 (31.8)	18 (27.3)		
Coronary, *n* (%)	18 (15.4)	18 (19.8)		15 (22.7)	13 (19.7)		
Valve, *n* (%)	29 (25.0)	43 (47.3)		25 (37.9)	30 (45.5)		
Other procedures, *n* (%)	11 (9.4)	6 (6.6)		5 (7.6)	5 (7.6)		
Complex, *n* (%)	22 (18.8)	26 (28.6)	.13	18 (27.3)	20 (30.3)	.84	0.067
Operative time (minute)	379 [316-461]	309 [276-408]	.004	350 [306-425]	310 [278-444]	.281	0.101
CPB time (minute)	180 [147-216]	147 [126-195]	.001	169 [140-192]	156 [128-212]	.745	0.028
Intraoperative fluid balance (mL)	2139 [566-3401]	1656 [239-3007]	.089	2016 [566-3401]	1582 [228-2928]	.157	0.18
Blood transfusion, *n* (%)	112 (95.7)	81 (89.0)	.11	61 (92.4)	59 (89.4)	.76	0.106
BG level at the start of surgery (mg/dL)	128 [106-153]	118 [104-132]	.016	118 [104-139]	118 [103-135]	.661	0.016
BG level at admission to ICU (mg/dL)	212 [191-232]	197 [176-218]	.004	205 [186-225]	198 [178-230]	.451	0.068
**Outcomes**							
SSIs	10 (8.5)	1 (1.1)	.039	8 (12.1)	1 (1.5)	.038	
Mediastinitis, *n* (%)	3 (2.6)	1 (1.1)	.79	2 (3.0)	1 (1.5)	>.99	
In-hospital mortality, *n* (%)	7 (6.0)	4 (4.4)	.84	3 (4.5)	2 (3.0)	>.99	
30-day mortality, *n* (%)	3 (2.6)	4 (4.4)	>.99	1 (1.5)	1 (1.5)	>.99	
Blood glucose levels							
24 hours mean (mg/dL)	194 [183-208]	148 [136-159]	<.001	195 [183-208]	149 [136-161]	<.001	
48 hours mean (mg/dL)	180 [167-197]	146 [135-157]	<.001	180 [168-198]	150 [136-160]	<.001	
Coefficient of variation (%)	3.9 [3.3-5.3]	3.7 [2.4-4.5]	<.001	4.0 [3.4-5.4]	3.8 [2.5-4.6]	<.001	
Time in range of target glucose (%)	48.0 [28.0-64.0]	88.0 [80.0-96.0]	<.001	53.1 [29.6-75.5]	88.0 [80.0-95.0]	<.001	
Time required to reach <180 mg/dL (hour)	9.0 [6.0-12.8]	3.0 [2.0-4.0]	<.001	8.5 [5.0-14.0]	3.0 [2.0-5.0]	<.001	
Hypoglycaemic events, *n* (%)	0 (0)	0 (0)	–	0 (0)	0 (0)	–	
Peak WBC (×10^3^/μL)	13.7 [11.9-17.4]	15.0 [12.2-17.5]	.265	13.4 [11.8-16.8]	15.0 [12.2-17.8]	.193	
Peak CRP (mg/dL)	9.7 [7.3-13.4]	8.4 [5.9-12.0]	.040	8.7 [6.2-12.8]	8.5 [5.9-12.1]	.873	

Continuous variables are expressed as median [interquartile range: 25th-75th percentile]. Abbreviations: AP = artificial pancreas; BG = blood glucose; CII = continuous insulin infusion; CPB = cardiopulmonary bypass; CRP = C-reactive protein; HbA1c = haemoglobin A1c; ICU = intensive care unit; SMD = standardized mean difference; WBC = white blood cell.

## Results

### Baseline characteristics

Before matching, the AP group had more males, complex surgeries, and longer operative times. Propensity score matching yielded 66 well-balanced pairs (Table 1).

### Glycaemic control

Control was significantly better in the AP group, reaching BG <180 mg/dL earlier than CII patients (median 3.0 vs 8.5 hour; *P* < .001). Over 48 hours, the AP group demonstrated lower mean BG (150 vs 180 mg/dL; *P* < .001), lower glycaemic variability (CV 3.8% vs 4.0%; *P* < .001), and higher TIR 70-180 mg/dL (88.0% vs 53.1%; *P* < .001) (Table 1). No hypoglycaemic events (BG <70 mg/dL) occurred in either group.

### Clinical outcomes

SSI incidence was significantly lower in the AP group (1.5% [1/66] vs 12.1% [8/66]; *P* = .038). Peak white blood cell, C-reactive protein, 30-day mortality, and in-hospital mortality did not differ significantly between groups post-matching.

## Discussion

Our study suggests that the automated AP system is associated with superior glycaemic control and a significantly lower SSI incidence compared with conventional CII. Postoperative hyperglycaemia impairs innate immunity; by achieving faster glucose normalization, the AP may mitigate this vulnerability. Its ability to maintain higher TIR with low variability likely contributes to sustained metabolic stability.

The NICE-SUGAR trial highlighted that strict control increased mortality due to iatrogenic hypoglycaemia.^9^ Manual CII, dependent on intermittent sampling, is inherently reactive and often inadequate for rapid post-operative metabolic shifts. Meanwhile, the AP system enables minute-by-minute closed-loop modulation, achieving tight control without hypoglycaemic events in our cohort while likely reducing nursing workload. Economically, European data suggest severe SSIs add approximately €23 000 per case to hospital expenditures.^10^ By reducing these costly complications, the AP system may offset its operational costs.

This study has limitations, including its retrospective single-centre design and small matched cohort. Differences in target ranges and monitoring frequencies, alongside our conservative CII protocol, complicate direct comparisons and may introduce bias. Residual confounding cannot be excluded, rendering these findings hypothesis-generating. Nevertheless, the observed reduction in SSIs highlights the potential clinical utility of the AP system. By mitigating manual constraints and hypoglycaemia risks, the AP may translate precise glycaemic control into tangible clinical and economic benefits.

## Conclusion

The automated closed-loop AP system safely achieves faster, more stable glycaemic control than conventional CII. Furthermore, AP use is significantly associated with reduced SSI incidence. Despite the observational design and differing protocol targets, our findings highlight the AP system’s potential to improve cardiac surgical outcomes, warranting validation in prospective multicentre trials.

## Data Availability

The data supporting the findings in this article are available from the corresponding author upon reasonable request.
